# Optimization of Culture Conditions for Amoxicillin Degrading Bacteria Screened from Pig Manure

**DOI:** 10.3390/ijerph17061973

**Published:** 2020-03-17

**Authors:** Xuanjiang Yang, Panpan Guo, Miao Li, Hualong Li, Zelin Hu, Xianwang Liu, Qiang Zhang

**Affiliations:** 1 Institute of intelligent machinery, Hefei Institute of Material Sciences, Chinese Academy of Sciences, Hefei 230031, China; xjyang@mail.ustc.edu.cn (X.Y.); panpan2019520@163.com (P.G.); hlli@iim.ac.cn (H.L.); zlhu@iim.ac.cn (Z.H.); lxw440@163.com (X.L.); 2 Department of Biosystems Engineering, University of Manitoba, Winnipeg, MB R3T 5V6, Canada; Qiang.Zhang@umanitoba.ca

**Keywords:** amoxicillin, biodegradation, Plackett–Burman, Box–Behnken design, culture condition

## Abstract

(1) Objective: The objective of this study was to screen amoxicillin (AMX)-degrading bacterial strains in pig manure and optimize the fermentation conditions for these strains to achieve high fermentation rate, which can provide an effective way for the practical application of bacterial strains as antibiotic-degrading bacterial in treating livestock waste for antibiotic residues. (2) Methods: Antibiotic susceptibility tests and high-performance liquid chromatography tandem mass spectrometry (HPLC-MS/MS) were employed to screen AMX-degrading bacterial strains in pig manure. The culture conditions were optimized for AMX-degrading bacterial strains using Plackeet–Burman design (PBD), the steepest ascent design, and the response surface methods, coupled with the Box–Behnken design (BBD). The effects of culture time, temperature, rotator (mixing) speed, inoculum level, and initial pH value on the growth of AMX-degrading strains were investigated. Experimental data obtained from BBD were utilized to generate a second-order polynomial regression model for evaluating the effects of the tested variables on the optical density at 600 nm (OD_600_) of culture solutions as the growth indicator for the screened AMX-degrading strains. (3) Results: The initial pH, culture time, and the inoculum level had significant effects on the OD_600_ value (growth) of the screened AMX-degrading strains. The initial pH value was found to be the most critical factor influencing the growth of bacteria. The optimized culture condition for the bacterial growth determined by the response surface methodology was: the initial pH of 6.9, culture time of 52 h, and inoculum level of 2%. The average OD value of 12 different fermentation conditions in the initial fermentation tests in this study was 1.72 and the optimization resulted in an OD value of 3.00. The verification experiment resulted in an OD value of 2.94, which confirmed the adequacy of the optimization model for the determining the optimal culture condition. (4) Conclusions: The growth of the screened strain of AMX-degrading bacteria could be optimized by changing the fermentation conditions. The optimization could be achieved by using the Box–Behnken response surface method and Plackett–Burman experimental design.

## 1. Introduction

Amoxicillin (AMX) is a broad-spectrum therapeutic antibiotic that can penetrate the cell walls of bacteria and thus kill pathogenic G^+^ and G^-^ bacteria (including coccus and bacillus) [1]. AMX has been commonly used in intensive livestock production because it is considered to be effective in treating a variety of animal diseases. However, like most antibiotics, AMX cannot be completely decomposed in the animal body and residues are excreted in the form of protoplasts or metabolic intermediates, which may eventually enter the environment [2]. AMX residues discharged into the soil and groundwater environment are harmful to ecosystems [3,4].

Unlike other natural penicillin antibiotics, AMX is acid-stable and difficult to hydrolysis [5]. Compared to the chemical or the physical methods for treating AMX residues, the microbial degradation methods are effective and more environmentally friendly for removing AMX residues in animal wastes. High-catalytic-activity enzyme generated by metabolic processes of microorganisms can reduce antibiotic activity by modifying the structure of antibiotic directly or indirectly [6]. Therefore, screening AMX degradation bacterial strains and optimizing the fermentation rate for these strains would potentially provide effective measures for degrading AMX residues in the environment. 

The rate of removing AMX by microbial degradation is highly dependent on the bacterial strains as well as their fermentation conditions. For a given strain, minor changes in culture conditions can impact the quality and quantity of the removal of AMX antibiotic residues [7]. However, the optimization of fermentation conditions of culture media is very tedious and complicated because many factors have to be considered [8]. The Response Surface Methodology (RSM) is an effective experimental method that can identify the optimal fermentation conditions for a multi-variable system mathematically and statistically [9,10]. The procedure of RSM to optimize the fermentation conditions may be briefly described as follows: (i) identifying factors that significantly impact the fermentation conditions using the Plackeet-Burman (PB) design method, which can identify significant factors among a larger number of influencing variables and eliminate relatively minor factors [11,12]; (ii) determining the change step size of each significant factor that are identified by PBD using steepest ascent design, which can confirm the optimal value area quickly and economically [13]; (iii) establishing a fermentation model and determining the optimal fermentation conditions by performing the Box-Behnken design (BBD), which reduces the number of experimental runs and provides enough information for the interaction between independent variables [14]; and (iv) comparing the predicted response value directly obtained by the BBD design with the experimental response value obtained by the optimal fermentation conditions, so as to verify the adequacy of the model. The RSM has been widely used on optimizing microbial fermentation conditions to avoid the unnecessary trails and addition of excessive components in the culture medium. For example, the RSM was used for optimizing the operation parameters on the photocatalytic degradation of chloramphenicol (CAP) [15] and to investigate the effects of processing factors in the preparation of antioxidant peptides hydrolyzed from goat’s milk [16]. The objective of this study was to first screen AMX-degrading bacterial strains in pig manure and then optimize the fermentation conditions for these strains to achieve high fermentation rate.

## 2. Materials and Methods

The experiments were performed to: (i) isolate efficient AMX degrading bacterial strains from pig manure using antibiotic susceptibility test and HPLC-MS/MS method; and ii) optimize the fermentation conditions of isolated bacterial strains by using the RSM method to provide a theoretical basis for industrial production of antibiotic degrading bacteria.

### 2.1. Isolation and Selection of AMX-Degrading Bacterial Strains

AMX-degrading bacterial strains were isolated and screened from the pig manure that was collected from a pig farm in Hefei, Anhui, China. This farm was selected because AMX residues were found in pig manure, which naturally led to the emergence of bacteria that feed on AMX. A total of 1000 g of pig manure was gathered from the farm. Collected pig manure was placed in sealed sterile bags and kept at a temperature about −20 °C before the experiment. Isolating and screening AMX-degrading bacterial strains were performed in three steps, namely enrichment, acclimatization and separation. The details of the steps can be found in the previously published reference [17].

Evaluating the degrading efficiency of bacterial strains is the key in bacterial screening. The antibiotic susceptibility test and HPLC-MS/MS methods were carried out to evaluate the degradation efficiency. The antibiotic susceptibility test qualitatively determined if an isolated bacterial strain was capable of degrading AMX. In this test, a piece of filter paper containing 10 μg of AMX was applied to the surface of the Luria–Bertani (LB) agar medium (NaCl (5 g/L), yeast extract (5 g/L), 1.5% (w/v) agar powder and tryptone (10 g/L) at pH 7.0) which had been inoculated with a degradation bacterial strain. As the diffusion distance of AMX in the agar increased, the AMX concentration decreased logarithmically to a certain concentration below which the bacterium would not grow, thus forming a transparent antimicrobial circle on the filter paper. The size of this inhibition zone reflected the sensitivity of the test bacterial to AMX, i.e., the smaller the circle, the stronger resistance of bacteria to AMX. The HPLC-MS/MS method was then used on the bacteria strains that had the small inhibitory zones observed in the susceptibility tests to further evaluate quantitatively their effectiveness in degrading AMX.

A Liquid chromatography system-UPLC (I-Class)-MS (XEXO TQD) (Waters, Milford MA, US) coupled with Themo LENGEND MICRO 17R (Thermo Fisher Scientific, USA) were used in the HPLC-MS/MS evaluation. The Waters ACQUITY UPLC BEH-C18 column (1.7 µm, 2.1 × 100 mm; Waters, USA) with column temperature of 35 °C was used. A mobile phase of 0.1% (v/v) formic acid in water (A) and 0.1% (v/v) formic acid in acetonitrile (B) was used, filtered through a 0.22 µm membrane filter at a flow rate of 0.4 mL/min. The gradient dilution was performed as follows: 0 min, 95% A; 1 min, 95% A; 3 min, 2% A; 5 min, 2% A; 5.5 min, 95% A; 8 min, 95% A. The injection volume was 1 µL for both samples and standards. The mass spectrometer was used for the detection of analytes in the positive ion mode. The ionization source used a source voltage of 3.00 KV; a source temperature of 400 °C; a gas flow rate of 700 L/Hr; and cone of 50 L/Hr.

The standard stock solution of AMX was prepared with ultrapure water at a concentration of 1 μg/mL, which was then used to dilute to the following concentrations: 500, 200, 100, 50, 20, 10, 5, 2, and 1 ng/mL. The culture medium samples containing the isolated bacteria strains was diluted 1000 times and centrifuged for 10 min at 13,000 rpm. The supernatant was removed and filtered through a 0.22 µm membrane and aliquots of the supernatant were taken for HPLC-MS/MS analysis. The isolated strains that had higher degradation rates were selected for the optimization of fermentation conditions.

### 2.2. Optimization of Fermentation Condition

#### 2.2.1. Determination of the Growth Curve of Bacteria Strain

The isolated bacteria strains were inoculated on LB liquid medium at 1% inoculation level and cultured at 30 °C for 24 h on a shaker incubator. A turbidimetric method was used to evaluate the growth curve of the bacteria [18,19], in which the growth of the bacteria was measured by the optical density of the bacterial cultural media at 600 nm (OD_600_) [20] using 722 ultraviolet visible light spectrophotometer (Thermo Fisher Scientific, Waltham, MA, USA) in 4, 6, 12, 15, 25, 35, 37, 40, 48, 50, 55, and 60 h of culture. The bacterial growth curve was plotted as the optical density OD_600_ vs. the culture time.

#### 2.2.2. Fermentation Experiment

The fermentation experiment was conducted to examine the effects of fermentation conditions on bacterial growth. Bacterial suspensions were inoculated on 50 mL LB liquid medium and cultured in a shaker incubator. The range of fermentation conditions (the influencing factors and levels) was selected as follows: the fermentation time (20, 30, 40, 50, and 60 h), temperature (20, 25, 30, 35, and 40 °C), the inoculum level (0.5%, 1%, 1.5%, 2.0%, and 2.5%), the shaker rotation speed (110, 120, 130, 140, and 150 rpm), and pH (5, 6, 7, 8, and 9) [21,22].

#### 2.2.3. Selection of Significant Variables by Plackett-Burman Design (PBD)

Based on the data from the fermentation experiment, the Plackeet–Burman design (PBD) was used to screen the factors that have the most significant influence among a set of factors by using a minimum number of tests [11]. It should be noted that this design is not focused on the interrelationship between factors, but merely on the significant effects of these factors [12]. Specifically, the effects of five parameters that defined the culture condition on the OD_600_ value of bacterial cultures were investigated. The number of tests selected was N = 12, with five parameters X_1_, X_2_, X_3_, X_4_, and X_5_ representing the fermentation time, the temperature, the inoculum level, the shaker speed, and pH, respectively. The high (+ 1) and low (- 1) levels of these parameters are shown in Table 1, with the high level being 1.25 times the low level. The regression analysis was carried out using the software MINITAB 17.0 (Minitab, LLC, State College PA, USA) to assess the statistical significance.

#### 2.2.4. Steepest Ascent Design (SAD)

Three most significant influencing variables selected by the PBD were further analyzed by the steepest ascent design (SAD) to design the next experiment (selecting the rage of parameters) to further optimize the fermentation condition. The steepest ascent design uses the gradient direction of the experimental data as the climbing direction, and the change step size is determined according to the effect value of each factor, which can approach the target area quickly and economically [13].

#### 2.2.5. Box-Benhnken Design (BBD)

The three significant factors and the best level of these factors identified after PBD and SAD were further optimized using Box-Behnken response surface design (BBD) [14]. This design coupled with the response surface methodology (RSM) is one of the most commonly used methods for process optimization with minimal experimental requirements [23]. In this design, the optical density (OD_600_) value (growth) of bacteria was considered as the response value and the three identified significant variables the independent variables. Data analysis and experimental design were performed for each single factor using Design-Expert (Version 8.0.6, Stat-Ease Inc., Minneapolis, MN, USA).

Based on the BBD, a set of 12 tests were performed and a second-order polynomial model was used to fit to the data to generate a prediction model for the optimal fermentation condition.

Y = α0 +α1Xm + α2Xn+ α3Xp + α11Xm2 + α22Xn2 + α33Xp2 + α12XmXn + α13XmXp+ α23XnXp(1)

where Y is the response (bacterial growth), X_m_, X_n_ and X_P_ are the three most significant parameters identified by the PBS among the five factors that were tested in the first set of fermentation tests (Table 1); α_0_ is the offset term, and α_i_, α_ii_ and α_ij_ are the regression coefficients of the first-order main effect, the second main effect, and interaction effect, respectively.

#### 2.2.6. Verification

To verify the effectiveness of the three-step optimization procedure (PBD–SAD–BBD), three separate tests were carried out according to the optimal fermentation conditions identified through the BBD experiments. The result was compared to the values predicted by the optimization Equation (1).

## 3. Results and Discussion

### 3.1. Isolation and Preliminary Screening of AMX- Degrading Bacterial Strains from the Pig Manure

**Figure 1:**
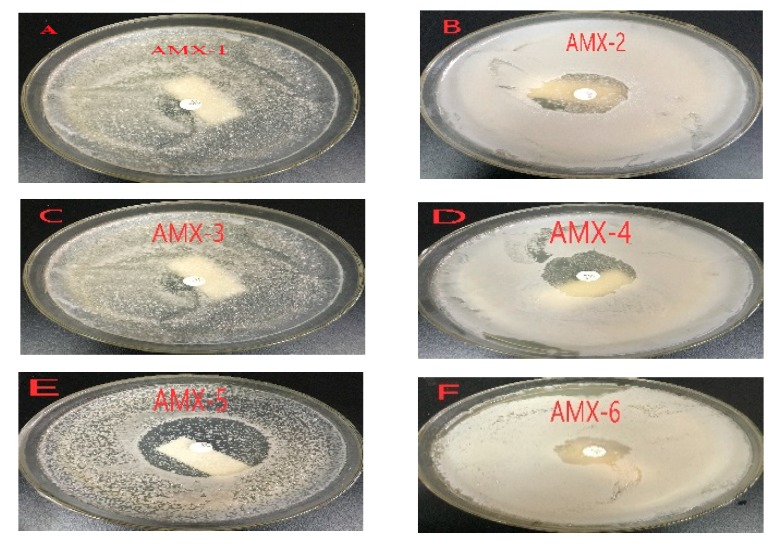
Results of amoxicillin (AMX) susceptibility test of six bacterial strain isolated from pig manure: (**A**) AMX-1; (**B**) AMX-2; (**C**) AMX-3; (**D**) AMX-4; (**E**) AMX-5; (**F**) AMX-6.

A total of six AMX-degrading bacterial strains (denoted as AMX-1, AMX-2, AMX-3, AMX-4, AMX-5, AMX-6) capable of growing on AMX as sole carbon and energy source were isolated from the pig manure. From the antibiotic susceptibility test, the inhibitory zone diameters of AMX-1, AMX-2, AMX-3, AMX-4, AMX-5, AMX-6 were determined to be 6.52 ± 0.28 mm, 16.85 ± 2.13 mm, 8.93 ± 1.24 mm, 21.52 ± 1.87 mm, 36.87 ± 1.48 mm and 10.75 ± 1.24 mm (Mean ± standard deviation, SD) (Figure 1), respectively. The inhibitory zone diameters of AMX-2, AMX-4 and AMX-5 were significantly larger than that of the other three strains (AMX-1, AMX-3, and AMX-6) (*P < 0.05*), and there were no statistically significant differences among AMX-1, AMX-3, and AMX-6. Therefore, AMX-2, AMX-4 and AMX-5 were considered to be less effective in degrading AMX antibiotic, and the other three bacterial strains (AMX-1, AMX-3 and AMX-6) were selected for further quantitative evaluation by HPLC-MS/MS.

### 3.2. Screening by HPLC-MS/MS

**Figure 2:**
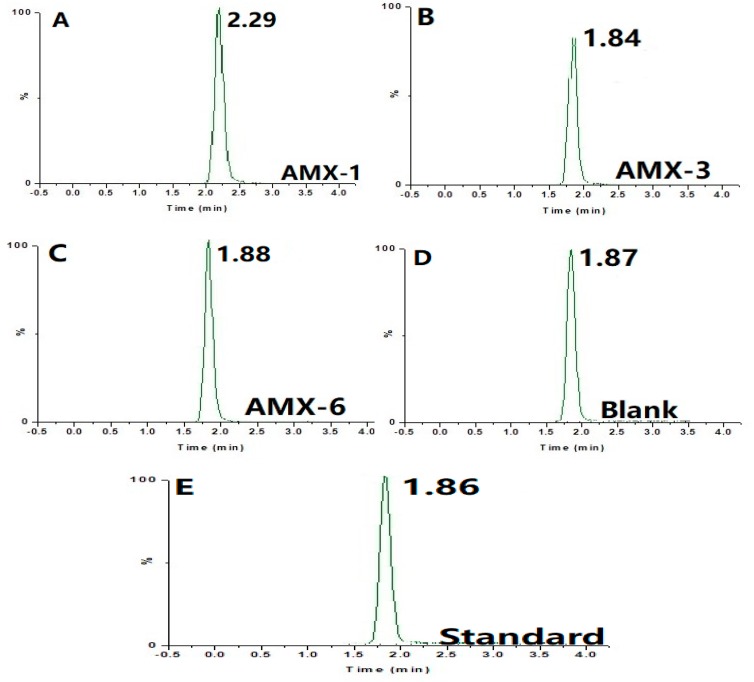
Results of HPLC-MS/MS analysis 90 min after inoculation of (**A**) strain AMX-1, (**B**) AMX-3, (**C**) AMX-6, (**D**) blank samples, (**E**) standard (non-inoculated) sample of AMX of 100 mg/L.

**Figure 3:**
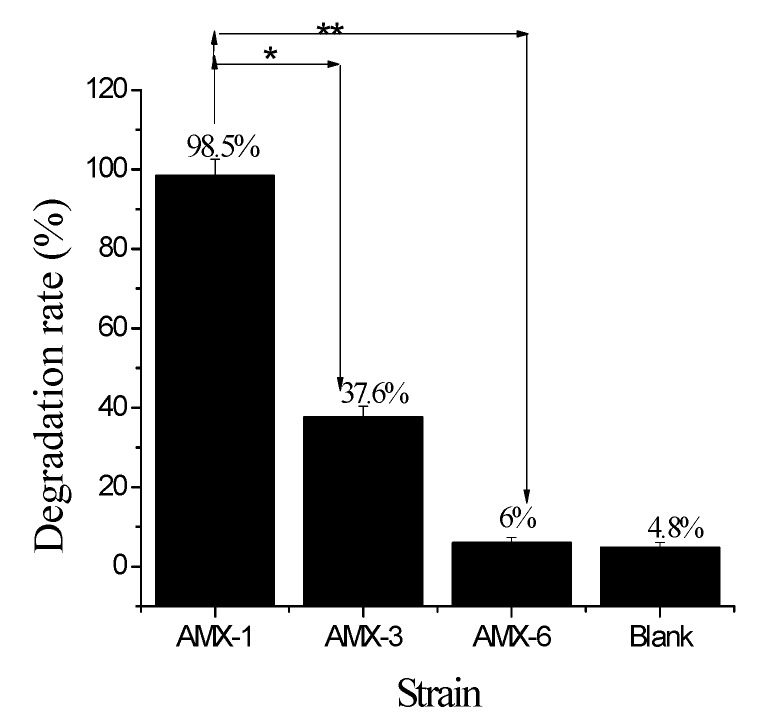
Comparison of AMX degradation by isolated bacterial strains. * indicates a significant difference at *p < 0.05*, and ** significant difference at *p < 0.01*.

The AMX degradation rates of strains AMX-1, AMX-3, and AMX-6 measured by using HPLC-MS/MS, along with the blank control, are summarized in Figure 2 and Figure 3 and Table 2.

The peak time of non-inoculated AMX standard solution detected by HPLC-MS/MS was 1.86 min (Figure 2E). Ninety (90) min after inoculation, AMX was still detected in the solutions of strains AMX-3 and AMX-6, but not in AMX-1 (a new unknown substance with an absorption peak at 2.29 was detected, Figure 2A). In other words, AMX was degraded and new degradation products were produced under the action of AMX-1 strain [24]. Regression analysis yielded a linear relationship between the concentration of AMX and the corresponding peak: Y = 39.31X − 1.71, R^2^ = 0.999. Using this relationship, the degradation rate by AMX-1 was calculated be to 98.5%, which was significantly higher than the other two strain (Figure 3). Therefore, AMX-1 bacterial strain was selected for the optimization of fermentation conditions.

### 3.3. Growth Curve of AMX-1

**Figure 4:**
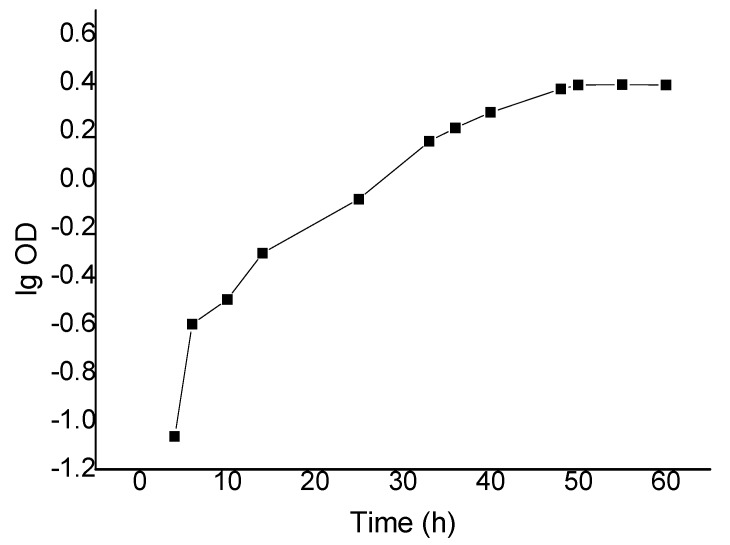
Growth curve of strain AMX-1.

The growth of AMX-1 strain roughly followed a typical “S” curve (Figure 4). A lag period occurred from 0 to 25 h, followed by a logarithmic growth period from 25 to 50 h, where the number of live bacteria increased rapidly and the maximum growth rate was reached. The growth reached a stable state after 55 h, which indicated the total number of living bacteria reached the maximum.

### 3.4. Effects of Individual Factors

**Figure 5:**
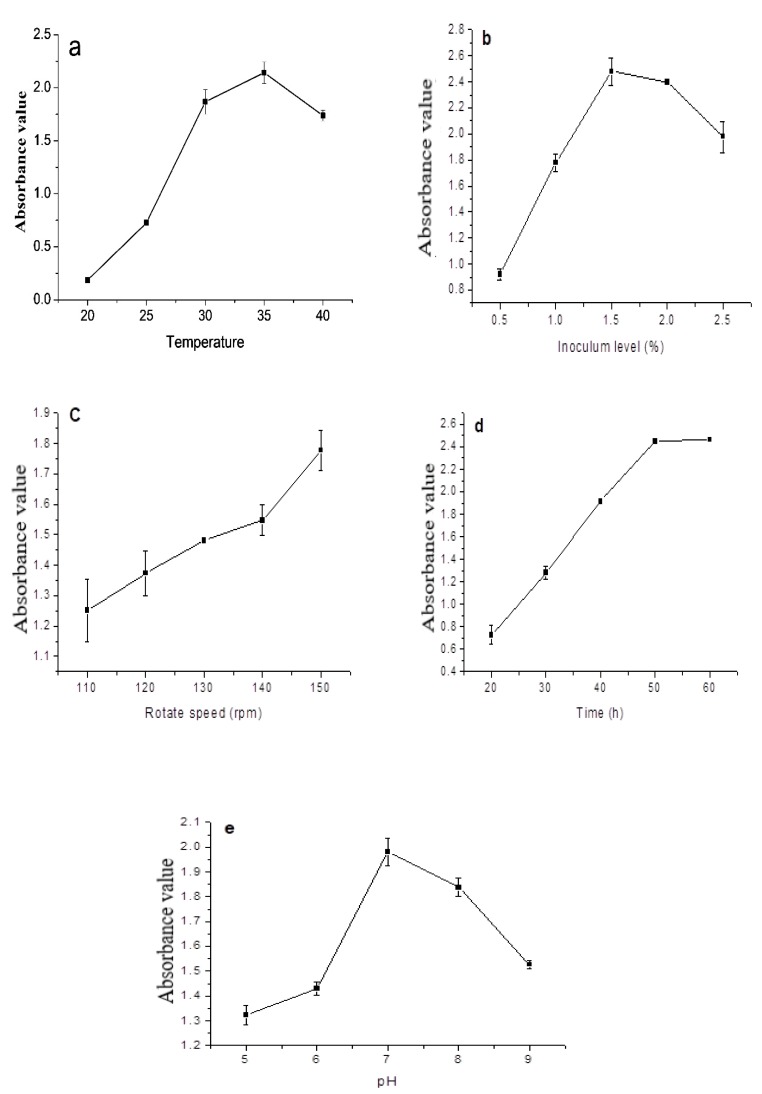
Results of the single-factor test. Effect of (**a**) temperature, (**b**) inoculum level, (**c**) rotate speed, (**d**) time, and (**e**) pH on the optical density (OD_600_) value of bacterial.

The measured optical density (OD_600_), representing the bacterial growth rate, increased first with temperature until about 35 °C and then decreased, with the quickest increase between 25 and 30 °C (Figure 5a). It was interesting to note that when the temperature was 40 °C, the OD_600_ value was even lower than that at 30 °C. It was clear that the optimal temperature range for AMX-1 growth was 30–35 °C, while other condition parameters were at the 0-level as described in Table 1.

The bacterial growth increased with the inoculum level rapidly between 1.0–1.5% (v/v), reaching the maximum at about 1.5% (v/v) (Figure 5b). Further increase in inoculum level resulted in a slower growth because oxygen and nutrients available to the bacteria in the medium became limiting and accompanied by bacterial proliferation, affecting the production of primary and secondary metabolites [25].

The shaker speed was related to the dissolved oxygen content in the culture solution [26,27]. Through the vibration of the shaker, the oxygen in the air was continuously dissolved into the culture solution for the bacteria. The bacterial growth increased with the rotation speed of the shaker linearly, indicating that the bacterial growth was closely related to the aeration level (Figure 5c), and that the bacterial strain was aerobic.

The bacterial growth increased with time and reached a maximum value at 50 h (Figure 5d). The growth stayed almost constant when the time was extended to 60 h, indicating that the bacterial growth had entered a stable state after 50 h. The possible reasons were: (i) depletion of nutrients in the medium; (ii) imbalance of nutrients, such as the inappropriate C/N ratio; (iii) accumulation of harmful metabolites such as acid, alcohol, toxins, or hydrogen peroxide (H_2_O_2_); and (iv) physical and chemical conditions such as pH and redox potential becoming less suitable for the bacteria [28,29].

The bacterial strain grew well only in a narrow range of pH, with the optimal pH around 7 (Figure 5e). In the acidic or alkaline environment with a pH of 5 or 10, the OD_600_ value of this strain decreased significantly, which indicated that the initial pH value could significantly affect the growth of this strain.

### 3.5. Plackett–Burman Design (PBD) Screening

The purpose of PBD was to identify which conditions of fermentation had a significant effect on the growth of the AMX-1 strain. The optical density OD_600_ ranged from 1.589 (run 7) to 1.899 (run 9), as measured in the fermentation experiment (Table 3). The results of statistical analysis on the main effect are presented in Table 4. The significance of each factor was assessed using the *P-*value at the significance level of 0.05.

The significance of the five variables in terms of their effects on the optical density (OD_600_) was ranked as: X_1_ (Time) > X_3_ (Inoculum level) > X_5_ (pH) > X_2_ (Temperature) > X_4_ (Rotation speed). Among them, the effects of the fermentation time, inoculum level and pH were statistically significant (*P < 0.05*). Therefore, these three variables were considered as the significant factors in the next experiment.

### 3.6. Steepest Ascent Design

The Coefficient Estimate of X_1_, X_3_ and X_5_ presented in Table 4 indicated that the fermentation time and the initial pH value exhibited positive effects and the inoculum level exhibited a negative effect. Based on these observations, the directions of the gradients for X_1_, X_2_, and X_4_ in the steepest ascent design were determined (Table 5). Specifically, the time and pH were gradually increased, while the inoculum level was decreased. The corresponding optical density (OD_600_) value first increased and then decreased (Table 5), with the maximum OD_600_ at the fermentation time 50 h, pH 6.5, and inoculum level 1.5% (v/v).

### 3.7. Optimization: Box-Behnken Design

Based on the data in Table 5 (the maximum OD_600_ at fermentation time X_1_ = 50 h, the inoculum level X_3_ = 1.5% (v/v), and pH X_5_ = 6.5, a BBD design was proposed to further refine the optimal fermentation condition (Table 6). The corresponding experimental results based on this BBD are shown in Table 7.

Design-Expert 8.0 was used to analyze the data in Table 7 and the following regression equation was obtained:

Y = 2.616 + 0.00625X_1_ + 0.11625X_3_ + 0.545X_5_ + 0.16X_1_ × X_3_ − 0.0225X_3_ × X_5_ − 0.363X_12_ + 0.087X_32_ − 0.3225X_52_ (R^2^ = 0.9195) (2)

where Y is OD_600_ (the response value), X_1_ is time (hr), X_3_ is the inoculum level (%), and X_5_ is the initial pH value.

The details of regression analysis are summarized in Table 8.

**Figure 6:**
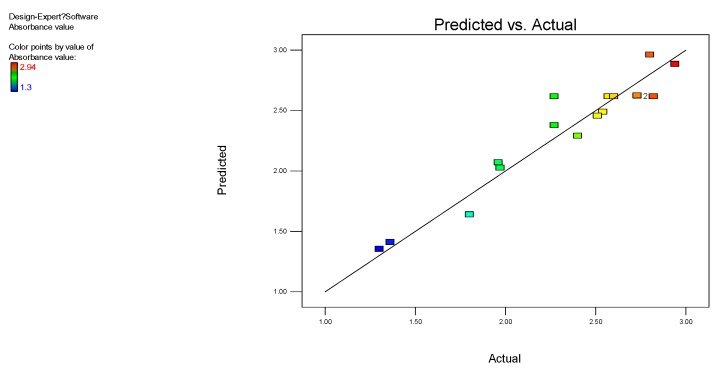
Internally studentized residuals.

The *P-*value for the overall model was 0.0044, which demonstrated that the quadratic equation model was highly significant [30]. Based on the *P-*values associated with the three factors, their influences on the optical density (OD_600_) of bacterial growth were ranked as: X_5_ (pH) > X_3_ (Inoculum level) > X_1_ (Time). The *P-*value for misfitting was 0.5733, indicating that the misfitting term was insignificant relative to the absolute error, and the misclassification is not significant. At the same time, the adjusted determination coefficient (Adj R-squared, Table 9) (0.82 > 0.80) and coefficient of variance (CV%) was 9.19, which further indicated that the multivariate regression relationship between the dependent variable and the all independent variables was significant [31]. In other words, the regression equation was adequate in predicting the optical density (OD_600_) under different fermentation conditions [32] (Figure 6).

#### Determination of Optimal Fermentation Condition

**Figure 7:**
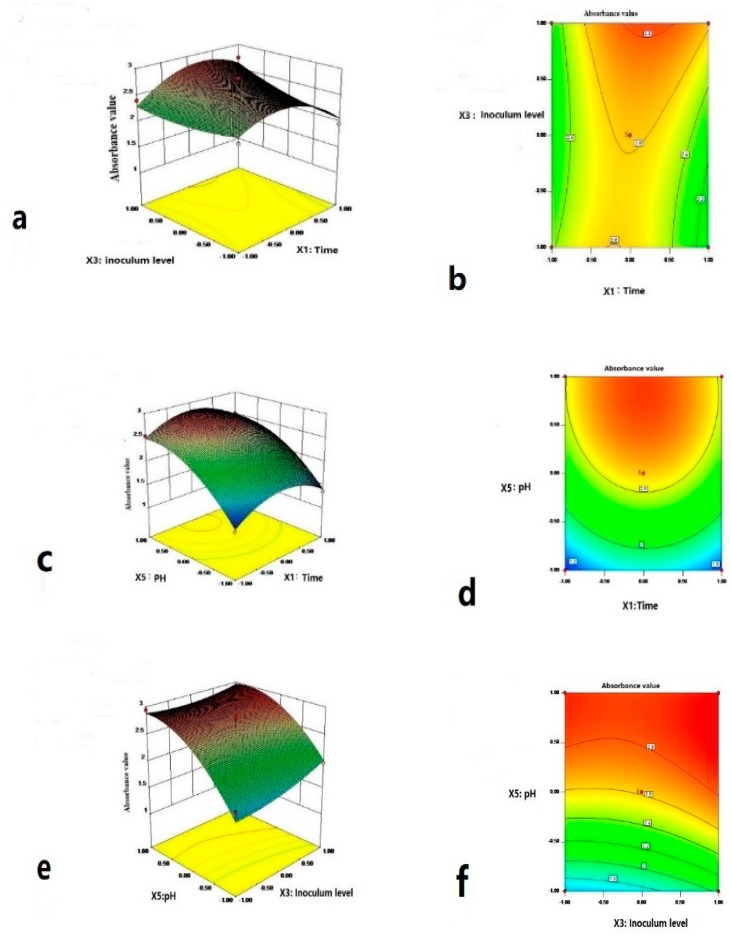
Response surface plots (3D) and contour plots (2D) for the effect of fermentation condition on OD_600_. (**a**,**b**) Effects of fermentation time and inoculum level; (**c**,**d**) effects of fermentation time and pH; and (**e**,**f**) effects of inoculum level and pH.

The response surface contour maps for the factors were generated based on the regression Equation (2) to search for the optimal fermentation condition (Figure 7).

The profiles of the response surfaces between fermentation time and inoculum level, fermentation time and pH, inoculum level and pH were all convex with an open downward direction, indicating a parabolic relationship between the OD_600_ value and the three factors of fermentation time, inoculum level and pH [33].The surface plots showed the interactive effects of the three significant fermentation factors on the bacterial growth. Figure 7b,c clearly demonstrates that the OD_600_ increased with pH, with the optimal pH value of 7.00 (Figure 7a) [29]. Comparing Figure 7a–c indicates that the pH also had the most significant effect on the bacterial growth among the three variables as the response surface curvatures were smaller (Figure 7a). Figure 7b,d, and m shows that the contour lines were circular, indicating the interaction of these three factors was not obvious [29,31]. Overall, the optimum fermentation condition for the screened bacterial strain was predicted to be: the fermentation time of 52.1 h, the inoculum level of 2%, and pH of 6.855, and this condition resulted in a predicted OD_600_ value of 3.00.

### 3.8. Experimental Verification

To verify the prediction model (Equation (2)), the predicted optimal fermentation condition (fermentation time X_1_ = 52 h, the inoculum level X_3_ = 2%, and pH X_5_ = 6.9) was used to conduct a set of tests to measure OD_600_. The average OD600 of five replicates was 2.94 with a standard deviation of 0.045. The difference between the predicted and measured values was about 2%, indicating that the model was adequate in predicting the optimal fermentation condition.

## 4. Conclusions

An effective Amoxicillin (AMX) degrading bacteria strain was successfully isolated and screened from pig manure through the antibiotic susceptibility testing and HPLC-MS/MS. A three-step procedure was developed to optimize the fermentation conditions for this screened bacteria strain. In the developed optimization procedure, the Plackett–Burman design (PBD) was first used to select most significant parameters; the steepest ascent design (SAD) was then used to define the gradient direction for the Box–Benhnken design (BBD); and finally the BBD with the response surface method (RSM) was used to refine the optimal fermentation condition, and a predictive model of quadratic polynomial was developed. It was found that among the five factors that defined the fermentation conditions, namely the inoculum level, the fermentation time, temperature, pH and shaker speed, three had significant effect on the growth of screened bacterial strain in the following order: pH, the fermentation time, and the inoculum level. The determined optimal fermentation condition was: pH 6.9, the fermentation time 52.1 h, and the inoculum level 2%. The proposed optimization process increased the bacterial growth rate by 67.6%. The proposed quadratic polynomial model adequately (within 2%) predicted the optimal fermentation condition.

## Figures and Tables

**Table 1:** Factors and levels of Plackett–Burman design (PBD).

Parameters	Level	
	−1	1
X_1_ Time (h)	40	50
X_2_ Temperature (°C)	30	37.5
X_3_ Inoculum level (%)	1	1.25
X_4_ Shaker speed (rpm)	120	150
X_5_ pH	6	7.5

**Table 2:** Regression equation model analysis.

Compound Name	Linear Range	Regression Equation	R^2^	Retention Time (min)
AMX	1–1000 ng/mL	Y = 39.31X − 1.71	0.9995	1.87

**Table 3:** Design and test results of Plackett–Burman.

Run Order	X_1_ Time (h)	X_2_ Temperature (°C)	X_3_ Inoculum Level	X_4_ Rotate Speed (rpm)	X_5_ pH	Optical Density
1	50	37.5	1.25	120	7.5	1.77
2	40	30	1.25	150	7.5	1.66
3	50	37.5	1	150	6	1.839
4	50	37.5	1	150	7.5	1.899
5	50	30	1.25	150	6	1.609
6	40	37.5	1.25	120	7.5	1.599
7	40	30	1	120	6	1.589
8	50	30	1.25	120	6	1.678
9	50	30	1	120	7.5	1.899
10	40	37.5	1	120	6	1.709
11	40	37.5	1.25	150	6	1.649
12	40	30	1	150	7.5	1.729

**Table 4:** Analysis of variance in Plackett–Burman.

Source	Sum of Squares	df	Mean Squares	F-Value	*P-*Value	Coefficient Estimate	Significance Ranking
Model	0.1173	5	0.0234	7.79	0.013		
X_1_	0.048	1	0.048	15.92	0.007	0.0633	1
X_2_	0.0076	1	0.0076	2.5	0.165	0.0251	4
X_3_	0.0407	1	0.0407	13.5	0.01	-0.0583	2
X_4_	0.0016	1	0.0017	0.55	0.487	0.0118	5
X_5_	0.0194	1	0.0194	6.45	0.044	0.0403	3
Residual	0.0181	6	0.003				
Cor total	0.1354	11					

**Table 5:** The design and results of steepest ascent test.

Test	Step Size	X_1_	X_3_	X_5_	OD_600_
1	X	30 h	2.5% (v/v)	5.5	1.48
2	X+ ΔX_i_	40 h	2.0% (v/v)	6.0	1.63
3	X+ Δ2X_i_	50 h	1.5% (v/v)	6.5	1.89
4	X+ Δ3X_i_	60 h	1.0% (v/v)	7.0	1.74
5	X+ Δ4X_i_	70 h	0.5% (v/v)	7.5	1.65

Note: ΔX_1_ = + 10 h, ΔX_3_ = −0.5% (v/v), ΔX_5_ = + 0.5.

**Table 6:** The factors levels in BBD.

Level.	X_1_ (Time) (h)	X_3_ (Inoculum Level) (%)	X_5_ (pH)
−1	40	1.00	6.00
0	50	1.5	6.5
1	60	2.0	7.0

**Table 7:** Experimental results of Box–Behnken Design.

Test	X_1_ Time (h)	X_3_ Inoculum Level (%)	X_5_ pH	Response Value (OD_600_)
1	−1	0	1	2.54
2	0	−1	1	2.94
3	0	0	0	2.27
4	1	1	0	2.73
5	−1	1	0	2.4
6	1	−1	0	1.96
7	0	0	0	2.57
8	1	0	1	2.51
9	0	−1	−1	1.8
10	0	1	1	2.80
11	1	0	−1	1.36
12	0	0	0	2.82
13	−1	0	−1	1.30
14	−1	−1	0	2.27
15	0	0	0	2.82
16	0	1	−1	1.97
17	0	0	0	2.60

**Table 8:** Analysis of variance of regression.

Source	Sum of Squares	df	Mean Square	F-Value	*P*-Value
X_1_	0.0003125	1	0.0003125	0.006794	0.9366
X_3_	0.11	1	0.11	2.35	0.1691
X_5_	2.38	1	2.38	51.66	0.0002
X_1_X_3_	0.1	1	0.1	2.23	0.1793
X_1_X_5_	0.002025	1	0.002525	0.044	0.8398
X_3_X_5_	0.024	1	0.024	0.52	0.4933
X_12_	0.55	1	0.55	12.06	0.0104
X_32_	0.032	1	0.032	0.69	0.4327
X_52_	0.45	1	0.45	9.7	0.017
Model	3.68	9	0.41	8.89	0.0044
Lack of Fit	0.12	3	0.039	0.76	0.5733
Pure Error	0.21	4	0.051		
Cor Total	4	16			

Note: The *P-*value less than 0.001 for the difference is extremely significant; the *P-*value less than 0.01 is highly significant; the *P-*value less than 0.05 is significant.

**Table 9:** Statistical significance.

Model Terms	Results
Std. dev.	0.21
Mean	2.33
%CV	9.19
PRESS	2.19
R-squared	0.9195
Adj R-squared	0.8161
Adeq precision	9.772
