# Erratum: Yang, X. et al. Optimization of Culture Conditions for Amoxicillin Degrading Bacteria Screened from Pig Manure. *Int. J. Environ. Res. Public Health* 2020, *17*, 1973

**DOI:** 10.3390/ijerph18020677

**Published:** 2021-01-14

**Authors:** Xuanjiang Yang, Panpan Guo, Miao Li, Hualong Li, Zelin Hu, Xianwang Liu, Qiang Zhang

**Affiliations:** 1 Institute of Intelligent Machinery, Hefei Institute of Material Sciences, Chinese Academy of Sciences, Hefei 230031, China; xjyang@mail.ustc.edu.cn (X.Y.); panpan2019520@163.com (P.G.); hlli@iim.ac.cn (H.L.); zlhu@iim.ac.cn (Z.H.); lxw440@163.com (X.L.); 2 Hefei Institutes of Physical Science, Chinese Academy of Sciences, University of Science and Technology of China, Hefei 230026, China; 3 Department of Biosystems Engineering, University of Manitoba, Winnipeg, MB R3T 5V6, Canada; Qiang.Zhang@umanitoba.ca

The second affiliation of the paper should have been included in our original article [1]. Therefore, in the section “Affiliation”, we would like to add the following information: The first author should also have been affiliated with the University of Science and Technology of China, because this affiliation plays an important role in providing the laboratory. Additionally, the order of Qiang Zhang’s affiliation should be changed accordingly.

The authors would like to apologize for any inconvenience caused to the readers by these changes.
